# Juvenile myoclonic epilepsy presenting as a new daily persistent-like headache

**DOI:** 10.1007/s10194-011-0363-z

**Published:** 2011-07-08

**Authors:** Todd D. Rozen

**Affiliations:** 1 Department of Neurology, Geisinger Health System, Wilkes-Barre, PA USA; 2 Geisinger Specialty Clinic, MC 37-31, 1000 E Mountain Drive, Wilkes-Barre, PA 18711 USA

**Keywords:** New daily persistent headache, Juvenile myoclonic epilepsy, Epilepsy, Headache

## Abstract

New daily persistent headache (NDPH) is a recognized subtype of chronic daily headache with a unique presentation of a daily headache from onset typically in individuals with minimal or no prior headache history. Various secondary mimics of NDPH have now been documented but at present there has been no association made between primary epilepsy syndromes and new daily persistent-like headaches. A case patient is presented who developed a daily continuous headache from onset who 3 months after headache initiation had her first generalized tonic-clonic seizure. Further investigation into her history and her specific EEG pattern suggested a diagnosis of juvenile myoclonic epilepsy (JME). Her NDPH and seizures ceased with epilepsy treatment. Clinically relevant was that the headache was the primary persistent clinical symptom of her JME before the onset of generalized tonic-clonic seizures. The current case report adds another possible secondary cause of new daily persistent-like headaches to the medical literature and suggests another association between primary epilepsy syndromes and distinct headache syndromes.

## Introduction

New daily persistent headache (NDPH) is a recognized subform of chronic daily headache in the second edition of the International Classification of Headache Disorders criteria (ICHD-2) [1]. It is unique in its presentation of a daily headache from onset typically in patients with minimal or no prior headache history. Various secondary mimics of NDPH have been documented including cerebral vein thrombosis, low or elevated cerebrospinal fluid pressure as well as sphenoid sinusitis [2]. In most instances, no secondary cause is found. The pathogenesis of NDPH is presently unknown and it has been recognized as one of the most treatment refractory headache types in specialty clinics. There has been no association made between secondary NDPH-like headaches and epilepsy. A patient is reported who developed daily headaches from onset and eventually was diagnosed with juvenile myoclonic epilepsy (JME). Once her epilepsy was controlled with medication, her headaches ceased.

## Case report

A 19-year-old woman presented to a headache specialty clinic complaining of a daily headache for 3 months. She awoke one morning with the headache and it had been continuous since that time. She denied any triggering event surrounding headache onset including no flu-like illness, stressful-life event or surgical procedure. The only possible trigger was a change in her birth control prescription 1 month prior. Her previous headache history was very minimal with infrequent tension-type headaches (TTHs), although she had several family members with probable migraine. The current headache was bifrontal and temporal in location. Average daily pain intensity was severe 7–8 out of 10 on VAS scale. The pain was sharp and throbbing in quality and was constant with no pain-free moments. There were no aura-type spells. Associated symptoms included nausea, vomiting, photophobia, phonophobia, osmophobia, and lightheadedness. She denied having any cranial autonomic symptoms with her headaches. There was a possible positional component to the headache being somewhat better while lying supine and in the morning hours but never any pain freedom. She also stated that over the past 3 months she had two spells of loss of consciousness in which the patient believed she fainted. There was no tongue biting or incontinence with these spells and she was unaware of any post-ictal confusion. However, there were no witnesses to these events. Her past medical history was significant for bipolar disorder. This was well controlled on carbamazepine. General and neurologic examinations were non-focal except for the presence of occipitonuchal and temporal tenderness to palpation bilaterally. Her mental status evaluation was normal. She had questionable cervical spine hypermobility on examination. She was taking ibuprofen on a daily basis without any benefit. She started the ibuprofen only after she developed the daily headaches. She had tried oral sumatriptan, but it was not effective. Prior to her evaluation at the headache clinic, she had a brain MRI with and without gadolinium which was read as a normal study; however, review of the imaging questioned loss of signal in the superior sagittal sinus. A diagnosis of probable NDPH was made, but further neuroimaging was suggested to rule out secondary mimics including an magnetic resonance venogram (MRV) and a magnetic resonance angiogram (MRA) of the intracranial and extracranial vessels. An EEG was also ordered for the loss of consciousness spells to make sure she was not having seizures. The patient was prescribed gabapentin as a preventive agent and was given salsalate, metoclopramide, baclofen and hydroxyzine as abortive agents. The following day she took gabapentin for the first time and added a metoclopramide tablet for nausea. Within minutes of taking these medications, the patient became giddy and after 10 min had a witnessed generalized tonic-clonic seizure lasting 8 min in duration with post-ictal confusion. She was brought to the hospital and there was seen by an epilepsy specialist. Further questioning of the patient and her mother revealed that for the past 3 months, the same time she was experiencing her daily headache, she had begun to have episodes of staring spells each lasting up to 10 s in duration and in which she would be non-responsive, have lip smacking and be picking at her clothes. These were occurring two times per week on average. In addition, she would experience occasional myoclonic jerks but these were very infrequent. Her EEG on hospital admission demonstrated 3–5 Hz generalized spike and polyspike wave discharges. MRV, MRA brain and neck were normal. Based on her clinical history of probable absence seizures, new generalized tonic-clonic seizures, myoclonic jerks and her EEG pattern, it was felt that this was most consistent with a diagnosis of JME. She was started on lamotrigine for the JME with a plan of tapering her off the carbamazepine which can worsen JME symptoms. On follow-up evaluation 3 weeks after hospital discharge and on a dose of lamotrigine 100 mg/day, she was both seizure free and headache free. She has now been followed for 9 months without any recurrent headaches or seizures remaining on the lamotrigine.

## Discussion

There is a known comorbidity between epilepsy and migraine [3, 4]. However, outside of migraine there is very little data on the association of other headache syndromes and epileptic disorders. The present case report now suggests a possible association between a specific form of epilepsy JME and another form of headache NDPH. This expands on the recently published manuscript looking at headache in patients with JME in a retrospective analysis from Germany [5]. In this study, headache was noted in 47 of 75 patients with JME. Migraine was noted in 31 individuals (20 migraine without aura, 11 with aura), 14 patients had migraine and TTH while 16 had TTH alone. The authors stated that the prevalence of migraine, especially with aura, is greater in the JME population than what is expected in a general population. In this study, no one was diagnosed as having NDPH but ten patients stated that their headaches preceded the symptoms of JME, as in the present case patient, and in addition six of the JME patients also had chronic headaches (5 migraine, 1 TTH). It is possible that an NDPH-like headache could have been part of this subgroup. The present case patient’s headaches met the ICHD-2 criteria for NDPH except for the presence of migrainous-associated symptoms [1] and of course the fact that there was a probable underlying secondary cause, so in this case the author suggests an NDPH-like secondary headache as a result of or associated with seizures in JME. Looking at the available descriptive studies of NDPH, migrainous symptoms are not only common in these patients, but they may be almost as prevalent as that seen in migraine itself. Suggested revised criteria for NDPH include the addition of migrainous symptoms as they are very prevalent in this condition [6]. As this patient had distinct migrainous-associated symptoms with her daily headaches, one must also consider with her family history of migraine that her past “TTHs” were in reality episodic migraine without aura. She then developed migraine-like headaches on a daily basis along with an underlying seizure disorder. It is known that patients with epilepsy and migraine develop post-ictal headaches that can resemble migraine.

JME is hallmarked by the presence of myoclonic jerks, generalized tonic-clonic seizures and less commonly absence seizures. It is an inherited condition but the mode of inheritance is not yet known and there appears to be incomplete penetrance and varying phenotypic presentations even within families with the similar genetic abnormalities [7]. Typical seizure triggers include sleep deprivation, stress, alcohol usage and photic stimulation. Diagnostic EEG findings include bilateral synchronous 4–6-Hz spike or polyspike and slow-wave discharges on interictal recordings. As the case patient’s headache started at the same time she began to show manifestations of JME and the headaches completely alleviated once the epileptic syndrome was controlled with lamotrigine suggests a direct cause and effect relationship between the two disorders. What is clinically interesting is that the headache was the primary persistent clinical symptom of her initial JME presentation and why she presented to a neurologist, while the myoclonus and staring spells were intermittent and much less predominant symptoms before the onset of generalized tonic-clonic seizures. It is important to note however and probably the issue in this present case is that before patients with JME have their first ever generalized tonic-clonic seizure they can show subtle myoclonic seizures that will go unrecognized by the patient or their family and also by non-epilepsy specialists, and only after the first major seizure and EEG tracing will the diagnosis be correctly made. There appears to be one other published case of daily persistent headache as the primary manifestation of an underlying seizure disorder. Ghofrani et al. [8] reported on a 9-year-old boy with nonconvulsive status epilepticus (absence status) who presented with a daily severe continuous headache and irritability but no other complaints and as in our case patient the headache ceased when the seizures were treated and the EEG normalized. However, unlike our presented case the young boy with absence status had the headache for only 1 day before diagnosis of his seizures and treatment initiation thus there was not a prolonged headache course and this would not have met criteria for NDPH. As the present case patient did experience absence seizures by history, it is certainly possible she at times had periods of absence status and may be this was a contributor to her daily headaches? Finally, an alternative explanation for the case patient’s NDPH-like headache is that it was the sum of post-ictal migraine like headaches from repetitive daily dialeptic and myoclonic seizures. A constant daily headache may have a secondary cause from epileptic seizures, especially in patients who have a history of interictal migraine. Ictal headache as the only manifestation of epileptic seizures is rare so when faced with the symptoms of both headache and seizure, ictal EEG recordings should be performed if possible to document the true pathogenic mechanisms of these episodes [9]. How JME or any seizure disorder can lead to a daily persistent headache is unknown. Epileptic syndromes could be inducing repetitive episodes of cortical spreading depression and thus headaches or causing a state of cortical excitation which in some manner causes head pain? [10]

The current case report adds another possible secondary cause of NDPH to the medical literature and suggests another association between primary epilepsy syndromes and distinct headache syndromes. The typical suggested evaluation for NDPH includes neuroimaging and laboratory testing alone [11]. EEG should now be considered for the work-up of NDPH especially in those patients with a family history of seizures, episodic myoclonus or unknown spells of loss of consciousness/syncope looking for JME or other primary epilepsy syndromes.
